# How Do Patients with Chronic Diseases Make Usage Decisions regarding Mobile Health Monitoring Service?

**DOI:** 10.1155/2019/1351305

**Published:** 2019-02-25

**Authors:** Fanbo Meng, Xiaofei Zhang, Xitong Guo, Kee-hung Lai, Xinli Zhao

**Affiliations:** ^1^School of Business, Jiangnan University, Wuxi 214122, China; ^2^Business School, Nankai University, Tianjin 300071, China; ^3^School of Management, Harbin Institute of Technology, 92 West Dazhi Street, Harbin 150001, China; ^4^Faculty of Business, The Hong Kong Polytechnic University, Kowloon, Hong Kong

## Abstract

**Objectives:**

The increasing population of patients with chronic diseases generates great challenge of chronic disease management. The occurrence of mobile health monitoring service (MHMS) is beneficial to chronic disease prevention and health promotion. The objective of this study is to investigate how patients with chronic diseases make usage decisions on MHMS.

**Study Design:**

A survey.

**Methods:**

213 respondents with chronic diseases were asked to rate their level of health severity, negative health emotions, and health uncertainty avoidance. SmartPLS was used to test the measurement model.

**Results:**

Of 213 research respondents, 159 of them have one chronic disease, while 54 have more than one such disease. Perceived health severity of patients with chronic diseases positively influences MHMS usage intentions, while negative health emotions do not. Health uncertainty avoidance strengthens the effect of health severity but weakens the effect of negative health emotions on MHMS usage intentions.

**Conclusion:**

Patients with chronic diseases have a unique decision-making process regarding MHMS usage in which their special health-related factors and tendencies play a critical role in determining behavioral intentions.

## 1. Introduction

With the rapid approach of the aging society, patients with chronic diseases are increasingly making up a considerable proportion of the global population [1]. This phenomenon has manifested many global social issues and generated great public health concern. According to a recent report by the World Health Organization, for instance, chronic diseases account for 60% of all deaths worldwide [2] and 85% of those in China [1]. Expenses incurred for chronic diseases comprise about 75% of the total healthcare expenses in the United States [3]. Patients with chronic diseases need constant healthcare throughout their lifetime compared with patients with acute diseases [4]. The traditional passive healthcare services have failed to meet the medical demands of patients with chronic diseases [5]. Therefore, reducing the negative effects of chronic diseases and alleviating the patients' problems have become the first priority of public health.

The health monitoring service is a widely used approach for chronic disease management [6]. As there is no effective treatment for chronic diseases, the monitoring of these diseases can enable patients to monitor the major health risk indicators and thereby enable them to self-manage their health conditions efficiently [7]. Since the mobile health (mHealth) service can provide continuous and ubiquitous health monitoring services at the individual level [8, 9], the mobile health monitoring service (MHMS) offers a pragmatic solution for patients with chronic diseases. However, there is a lack of knowledge on how this special group makes usage decisions on this monitoring service, practically and theoretically. Therefore, our research proposes the first research question: *how do patients with chronic diseases make usage decisions regarding the mHealth monitoring service?*

To explore how chronic disease patients make decisions on an MHMS comprehensively, this research focuses on the health-related factors. The reasons are twofold: (1) the current literature on this issue has, thus far, mainly adopted a technology acceptance perspective and has largely ignored the health-related factors [10, 11] and (2) owing to the long-term influence of the chronic diseases, such patients would exhibit special reactions to health-related affairs [12], which may influence their decision-making on an MHMS. Thus, exploring patients' decision-making from a health perspective will not only add to the current knowledge on health technology adoption but also provide a basic understanding of how chronic disease patients make health-related decisions.

Due to the long-term influences of chronic diseases, chronically ill patients generally experience many challenges for managing their health problems. Patients with chronic disease may be in negative emotions, such as anxiety and depression when facing their health issues [12]. These negative health emotions are expected to last for a longer time as the diseases cannot be easily treated. On the contrary, facing the new challenges associated with chronic diseases, chronic disease patients have to self-manage their health matters in their daily lives, such as the need to pay more attention to health conditions and the chronic diseases [13]. These experiences will induce them to have a highly severe perception of their health conditions. Therefore, this study draws on negative health emotions and health severity to manifest the physical and emotional effects of the chronic diseases on patients, respectively. Accordingly, the second research question of this research is as follows: *do the characteristics of patients with chronic diseases, i.e., negative health emotions and health severity, influence their decision-making on their usage of the mHealth monitoring service?*

Prior evidence has indicated that services or transactions through virtual channels based on information and communications technology (ICT) can provoke many uncertainties and potential risks [14]. Individuals possess different tolerances of uncertainty in their daily decision-making, which is determined by their uncertainty avoidance characteristics [15]. Accordingly, when they make health-related decisions, their health uncertainty avoidance can sharpen their decision processes. Therefore, to gain a better understanding of the role of negative health emotions and health severity in patients' usage of the MHMS, this research further explores the contingency role of health uncertainty avoidance. The third research question is as follows: *Are the effects of negative health emotions and health severity contingency on patients' health uncertainty avoidance?*

To address the aforementioned questions, a theoretical research model is developed, which is further empirically tested by a survey among patients with chronic diseases. In doing so, this research contributes to the extant literature in several aspects. First, although the diffusion of mHealth services has been widely explored in the past decades, most prior studies have taken a technology perspective and largely neglected the health-related factors. Our research is possibly one of the first to explore how users decide to use the mHealth service mainly from a health perspective. Second, by explicitly investigating the contingency role of health uncertainty avoidance, our findings shed light on the relative importance of the physical and emotional conditions for chronically ill patients' MHMS usage decisions. Third, our research focuses on a special group, i.e., patients with chronic diseases, and explores their special responses to mHealth services. We also contribute practical implications arising from this research to guide mHealth practitioners and users.

## 2. Materials and Methods

### 2.1. Study Design

**Figure 1:**
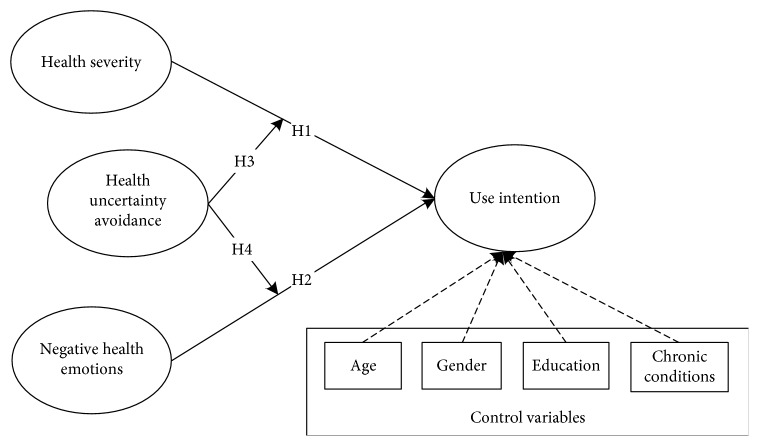
Conceptual research model.

To answer research questions, a survey was conducted among chronic disease patients in China by means of a questionnaire to test the research model (Figure 1). This questionnaire includes four sections: a brief introduction of the MHMS, their responses to the service (i.e., usage intentions), their perceptions of their own health conditions (i.e., health severity, negative health emotions, and health uncertainty avoidance), and their general information (i.e., age, gender, education, and chronic conditions). Adapted from the work of Johnston and Warkentin [16], health severity refers to the patients' perceptions of whether their health problems, i.e., chronic diseases, are serious issues. Based on the work of Anderson and Agarwal [17], negative health emotions refer to patients' emotions are closely related to their health conditions and the long-term chronic experiences generally lead patients to experience negative emotions such as anger, worry, and depression. Regarding the measurement of negative health emotions, it needs to mention that 4 items of 12 were selected in our study for two reasons. First, based on the results of a pilot study, using 12 items from negative health emotions makes the questionnaire so complicated and long that respondents will lose patience by filling the questionnaire. Second, these 4 items could also reflect the variable of negative health emotions in the pretest study. According to the work of Vishwanath [14], health uncertainty avoidance is defined as the tendency to avoid any unexpected or unknown risks and uncertainty in health-related decisions. As the theoretical constructs are widely measured and used in previous empirical studies, this research, therefore, adopts these measures and adapts them to our research context. The measures and their original sources are presented in Table 1.

### 2.2. Data Collection

The approval for this study was gained from the research ethics committee at our institution, and a written informed consent was obtained from all participants. Considering survey participants may have limited experiences and knowledge about the MHMS, we provided participants with a brief introduction to this new innovation. This MHMS could monitor patients' blood pressure readings, heart rates, blood glucose levels, etc. This service could provide alert condition signals related to physiological conditions. All participants were given US$ 3 as a form of motivation. The questionnaire was posted on a website. To minimize the selection bias, we recruited the participants using two approaches, i.e., by diffusing the links of the questionnaire in an online community for chronic disease patients and acquiring offline customers in a supermarket. Rather than restricting our study to patients with chronic diseases, we recruited the general population and used the questions regarding chronic conditions to obtain their information on health conditions so as to increase the reliability of their responses. A nonresponse bias test was conducted between these two groups using the method suggested by Armstrong and Overton [18]. Furthermore, the demographic information of both groups was tested, and no significant differences between them were found. A total of 504 participants completed the survey and thus were considered as valid participants. Among them, 213 experiencing one or more chronic diseases were our target respondents. To increase the validity of the research, we also analyze the respondents without chronic diseases to examine whether patients with chronic diseases respond differently to the MHMS compared with those without chronic diseases.

## 3. Results and Discussion

### 3.1. Results

Of our target research respondents, 159 of them have one chronic disease, while 54 have more than one such disease. Moreover, 59 of them suffer from chronic gastritis, 58 have hypertension, 37 have rheumatic arthritis, and 30 suffer from cardiovascular and cerebrovascular diseases. Of these, 40.4% are female. More than half of the respondents are in their forties, while 16.9% are in their fifties, and 3.8% are in their sixties. These age statistics are consistent with the age distribution of patients with chronic diseases in China [19]. To test our model, we conducted measurement model and structural model analyses subsequently.

SmartPLS was used to test our measurement model. The internal reliability, convergent validity, and discriminant validity of the measurement model were examined as indicators of the goodness of the measurement model. The reliability of the measurement model was assessed by examining Cronbach's alpha, composite reliability (CR), and average variance extracted (AVE) [20]. The results are presented in Tables 2 and 3.

In our study, the threshold values of CRs and AVE were 0.70 and 0.50, respectively, consistent with those in the study of Chin [21]. According to Nunnally [22], a value of at least 0.70 of Cronbach's alpha indicates adequate reliability. As perceived in Table 3, all constructs satisfied the criteria for reliability. Composite reliabilities for these constructs ranged from 0.822 to 0.938, and the AVE varied from 0.571 to 0.835. These results suggest that all indicators are above the cutoff values, indicating good construct reliability [20]. All the item loadings of each construct were significantly above the suggested cutoff value (0.700), indicating convergent validity [21]. All item loadings on expected constructs were greater than their cross loadings on other constructs, and the correlations of the constructs were significantly smaller than the square roots of the AVE values of each construct (Table 2), indicating that the constructs have good discriminant validity.

**Figure 2:**
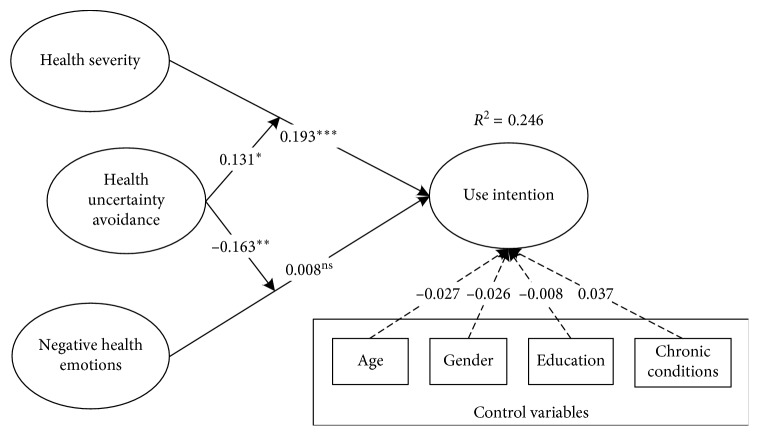
Results of the research model. ^*∗∗∗*^*P* < 0.01; ^*∗∗*^*P* < 0.05; ^*∗*^*P* < 0.1; ns: not significant.

The structural model was also analyzed by using the PLS. It was assessed by checking the significance of path coefficients (*β*) between various factors. First, the basic model without the moderating effects was tested. The results showed that negative health emotions have no significant effects on usage intentions (*β* = 0.008, *t* = 0.141) and health severity significantly influences usage intentions (*β* = 0.193, *t* = 3.372). The full model was then tested. The PLS results showed that except for the relationship between health negative emotions and usage intentions, all other proposed relationships were significant. Regarding the moderating effects, health uncertainty avoidance positively moderates the relationship between health severity and usage intentions (*β* = 0.131, *t* = 1.812). Health uncertainty avoidance negatively moderates the relationship between negative health emotions and usage intentions (*β* = −0.163, *t* = 2.416). These factors fully explain 24.6% of the variance of usage intentions. The PLS results of the structural model are recorded in Figure 2.

In particular, we tested the research model based on the data collected from respondents without chronic diseases. The results indicated that health severity had positive effects on usage intentions (*β* = 0.161, *t* = 2.825, *P* < 0.01). Negative health emotions have no significant effects on usage intentions (*β* = 0.042, *t* = 1.205, not significant). Health uncertainty avoidance strengthens the effects of health severity on usage intentions (*β* = 0.185, *t* = 2.960, *P* < 0.01). Therefore, these three hypotheses were consistent with the results of the research model based on the data collected from chronic disease patients. However, health uncertainty avoidance has positive moderating effects on the association between negative health emotions and usage intentions (*β* = 0.210, *t* = 2.639, *P* < 0.01), which is contrary to the result of the research model based on the data collected from respondents with chronic diseases. This controversial result may be due to the difference between the patients and respondents without chronic diseases. Without the diseases, when they perceive uncertainty about their health, they may believe the MHMS can help them, which weakens the negative effect of negative health emotion. These results show that the decision process regarding the MHMS is different among patients with chronic diseases and those without such diseases.

### 3.2. Discussion

By testing the proposed research model and hypotheses, our research highlights four aspects of the key findings. First, the health severity perceptions of patients with chronic diseases increase usage intentions of the MHMS for health monitoring. The rationale is that if chronic disease patients perceive their health problems as serious, they will feel a strong need to take health preventive measures, such as using the MHMS. This finding is consistent with prior studies that demonstrated health severity is a key predictor of health behavior [23, 24]. Second, chronic disease patients with negative health emotions were unlikely to use the MHMS. In contrast to those of earlier studies that indicated negative health emotions (e.g., worry and anxiety) are positively associated with health behavior [16, 25, 26], this controversial result seems acceptable given the special participants of our study. Chronic diseases are long-term conditions and cannot be cured easily and instantly, compared with the acute diseases [27]. Therefore, chronic disease patients may not experience negative health emotions as strong as patients with acute diseases, and thus they are unlikely to adopt MHMS as preventive measures. Third, health uncertainty avoidance strengthened the effects of health severity on usage intentions. Patients with highly serious health conditions will face many uncertainties regarding their physical conditions. In this situation, the high uncertainty avoidance will lead them to focus more on finding ways to reduce the uncertainties. Therefore, when making decisions on whether to use the MHMS for their chronic diseases, there is a higher possibility for them to choose usage to reduce their feelings of health uncertainty. Finally, health uncertainty avoidance weakened the effects of negative health emotions on usage intentions. This negative moderating effect indicates that chronic disease patients with negative health emotions have a higher tendency to make risk-seeking decisions to improve their current health conditions. However, those patients with high health uncertainty avoidance are more likely to choose more certain decisions toward their health conditions and thereby leading to lower use intention of the MHMS.

Our research contributes several implications to the extant literature. First, our study is possibly one of the first to explore the diffusion of the mHealth service from the perspective of health behavior theory. Although the diffusion of the mHealth or other services has been widely explored in recent decades, most prior studies have adopted a technology perspective and investigated the well-developed technology acceptance theories and models mainly from a theoretical lens. However, the health-related factors have been largely neglected. When exploring patients' responses to health services, their health severity and negative health emotions, as well as health uncertainty avoidance are critical predictors in determining their behavior. In this way, our study not only addresses the research gaps in the technology acceptance literature but also focuses on the role that patients' physical and emotional conditions play in their decision-making regarding new health-related services.

Second, this research explores the important contingency role of health uncertainty avoidance. As a characteristic of patients with chronic diseases, health uncertainty avoidance can play a significant role in their daily health-related decision-making. Our results indicate that health uncertainty avoidance positively moderates the effects of health severity and negatively moderates the effects of negative health emotions. The special contingency role of health uncertainty avoidance indicates that it plays a significant role in shaping patients' decision-making regarding health services. By explicitly investigating the contingency role of health uncertainty avoidance, our findings shed light on the relative importance of physical and emotional conditions for patients' mHealth usage decisions among different patient groups.

Third, this research provides a general picture of how patients with chronic diseases respond to the MHMS. Currently, more than 20% of the Chinese population is suffering from chronic diseases [28], and such diseases comprise 85% of total deaths and account for 70% of all medical expenses [29]. Furthermore, the MHMS is especially suitable for these patients. However, little research attention has been devoted to the MHMS usage behavior of this special group. By investigating the effects of their unique responses from their physical and emotional conditions, our research provides a basic understanding of how chronically ill patients respond to mHealth services.

By exploring how patients with chronic diseases respond to the MHMS from a health perspective, this study also provides insights into this group of patients and the service providers. Indeed, chronically ill patients need to be aware of the influences of their health severity perceptions and negative emotions on their health-related decisions. The serious health conditions will cause them to take more health preventive measures, without considering their health concerns. The tendency of avoiding health uncertainty also shapes their decision processes. Only by their awareness of such influences, are they able to make more rational decisions regarding protecting from their chronic conditions.

Service providers, on their part, should be aware of the factors that cause patients to use their services. As patients' serious perceptions on their health conditions positively influence their usage intentions, the providers are advised to exert efforts on informing the patients about the hazards of their chronic diseases. Hence, they can target their potential customers, the patients with serious chronic conditions, who have also realized the hazards of their diseases. In their role of facilitating health uncertainty avoidance, they can segment such customers in their marketing campaigns. To assist patients with serious health concerns, providers can assist them by facilitating health uncertainty avoidance, with measures such as paying more attention to those with a higher sense of health uncertainty avoidance. Providers can alleviate the problems faced by patients with negative emotions on their health status by devoting more attention to those with a lower sense of health uncertainty avoidance. In this way, the service providers will experience a higher possibility of transforming these patients with chronic diseases from potential customers to actual ones.

The increasing population of patients with chronic diseases has promoted the use of mobile ICTs in health monitoring services. While the MHMS is suitable for these patients, we know very little about how they make usage decisions regarding the MHMS, and whether their health-related features cause them to make different decisions. To address our research questions, this study creates a theoretical model to test the effects of health severity and negative health emotions on the usage intentions of the MHMS and the contingency role of health uncertainty avoidance. The model was tested by conducting a survey among patients with chronic diseases. We find that health severity positively influences usage intentions, while health uncertainty avoidance plays different moderating roles on the effects of health severity and negative health emotions. This research contributes to the understanding of the diffusion of the mHealth service, the role of health-related factors in decision-making, and the unique decision-making processes of patients with chronic diseases.

Limitations of this study deserve mention. First, chronic disease patients were taken as the sample in the study because this specific group accounts for a large portion of the whole mHealth services. The results of the study should be applied with caution when applying in other populations. Second, this study was conducted in China, which has a collectivistic culture. Thus, the results may be applicable only in cultural contexts similar to those of the Chinese mainland. Third, although the explanatory power of the model is acceptable (24.6% for usage intention), we still advocate the potential to enhance our explanatory power through taking additional factors into consideration, in future research.

## 4. Conclusions

The increasing population of patients with chronic diseases has promoted the use of mobile ICTs in mHealth monitoring services. While the MHMS is suitable for these patients, we know very little about how they make usage decisions regarding the MHMS, and whether their health-related features cause them to make different decisions. To address our research questions, this study creates a theoretical model to test the effects of health severity and negative health emotions on the usage intentions of the MHMS and the contingency role of health uncertainty avoidance. The model was tested by conducting a survey among patients with chronic diseases. We find that health severity positively influences usage intentions, while health uncertainty avoidance plays different moderating roles on the effects of health severity and negative health emotions. This research contributes to the understanding of the diffusion of the mHealth service, the role of health-related factors in decision-making, and the unique decision-making processes of patients with chronic diseases.

## Figures and Tables

**Table 1:** Research constructs and measurements.

Construct	Measurement	Disagreed (%)	Neutral (%)	Agreed (%)
Use intention [16]	I intend to use the MHMS in the next 3 months	6.9	17.9	75.2
	I predict I will use the MHMS in the next 3 months	7.9	16.9	75.2
	I plan to use the MHMS in the next 3 months	7.6	23.4	69

Negative health emotion [17]	I feel furious about my present health status	63.4	17.6	19.0
	My current health status is a real inconvenience	70.3	16.6	13.1
	My present health problems fill me with dread	38.6	21.7	39.7
	I feel disgusted with my current state of health	74.2	13.4	12.4

Health severity [16]	My health issues are severe	35.2	15.2	49.6
	My health issues are serious	45.2	15.9	38.9
	My health issues are significant	61.0	12.1	26.9

Health uncertainty avoidance [14]	If I use the MHMS, I will increase my effectiveness on avoidance of any uncertainty or unknown situations related to my health status	9.7	23.1	67.2
	If I use the MHMS, I will spend less time feeling concerned about any uncertainty or unknown situations related to my health status	7.9	19.7	72.4
	If I use the MHMS, I will improve the quality of avoidance of any uncertainty or unknown situations related to my health status	6.2	20.7	73.1
	If I use the MHMS, I will increase the quantity of output for the same amount of effort in avoiding any uncertainty or unknown situations related to my health status	5.9	20.3	73.8

*Note.* All items included in the survey were measured on a 7-point Likert scale ranging from 1 to 7, with “1” representing strongly disagree and “7” representing strongly agree.

**Table 2:** Loadings and cross loadings.

	BI	NHE	HSEV	HUAE
BI1	**0.904**	0.7	0.168	0.378
BI2	**0.906**	0.090	0.187	0.394
BI3	**0.931**	0.133	0.161	0.367
NHE1	0.023	**0.653**	0.380	0.002
NHE2	0.011	**0.679**	0.293	0.021
NHE3	0.168	**0.924**	0.361	0.112
NHE4	0.080	**0.735**	0.316	0.062
HSEV1	0.133	0.296	**0.779**	0.002
HSEV2	0.027	0.256	**0.679**	−0.003
HSEV3	0.188	0.382	**0.869**	0.039
HUAE1	0.328	0.079	−0.019	**0.845**
HUAE2	0.336	0.027	−0.030	**0.835**
HUAE3	0.410	0.123	0.094	**0.883**
HUAE4	0.315	0.108	0.029	**0.804**

*Note.* BI: behavioral intention; NHE: negative health emotion; HSEV: health severity; HUAE: health uncertainty avoidance.

**Table 3:** Correlations and discriminant validity.

	Cronbach's alpha	CR	AVE	BI	NHE	HSEV	HUAE
BI	0.901	0.938	0.835	**0.913**			
NHE	0.742	0.822	0.608	0.188	**0.779**		
HSEV	0.828	0.839	0.571	0.149	0.414	**0.755**	
HUAE	0.863	0.906	0.709	0.416	0.026	0.102	**0.842**

*Note.* The diagonally arranged bold numbers are the square roots of AVE values.

## Data Availability

The data used to support the findings of this study are currently under embargo, while the research findings are commercialized. Requests for data, after the publication of this article, will be considered by the corresponding author.
